# Genetic modulators of metabolic dysfunction-associated steatotic liver disease (MASLD) and their epistatic interactions: from in vitro and animal models to clinical outcomes

**DOI:** 10.1186/s12920-026-02332-7

**Published:** 2026-03-02

**Authors:** Fernanda G. Arriaga-González, Felipe de Jesús Castañeda-Córdova, Mauricio Díaz-Muñoz, Matthew Hoare, David J. Adams, Carla Daniela Robles-Espinoza, Christian Molina-Aguilar

**Affiliations:** 1 Laboratorio Internacional de Investigación sobre el Genoma Humano, Universidad Nacional Autónoma de México, Querétaro, Querétaro México; 2 Wellcome Sanger Institute, Hinxton, Cambridgeshire, CB10 1SA UK; 3 Instituto de Neurobiología, Universidad Nacional Autónoma de México, Querétaro, Querétaro México; 4 Early Cancer Institute, University of Cambridge, Cambridge, UK; 5 Department of Medicine, University of Cambridge, Cambridge, UK; 6 Cambridge Liver Unit, Addenbrooke’s Hospital, Cambridge, UK

**Keywords:** MASLD, Genetic risk alleles, Genetic epistasis, Protective alleles

## Abstract

Metabolic dysfunction-associated steatotic liver disease (MASLD), previously known as NAFLD (non-alcoholic fatty liver disease), is a growing global concern, affecting nearly a third of the world’s population. This umbrella term covers a range of liver pathologies, from reversible disease stages like simple steatosis, to irreversible conditions such as cirrhosis and hepatocellular carcinoma (HCC). MASLD, which may result from metabolic risk factors like obesity and type 2 diabetes, is projected to increase due to a rise in sedentary lifestyles. This review addresses genetic influences that predispose to disease development, including the role of risk-conferring and protective/preventive alleles. The epistatic relationships between genetic variants can significantly influence the development and progression of MASLD. Key genetic variants, such as those located in the *PNPLA3*, *TM6SF2*, and *MBOAT7* genes, often interact to exacerbate MASLD severity and play key roles in lipid metabolism and liver inflammation. For example, the co-expression of certain *PNPLA3* and *TM6SF2* variants increases the risk of advanced fibrosis and HCC. Some variants located in *HSD17B13* and *MTARC1* offer protective effects, reducing the risk of severe liver disease despite comorbidities such as obesity, and can mitigate the harmful effects of these risk alleles. Additionally, the potential of polygenic risk scores (PRS) to predict MASLD development and its complications is also discussed, although challenges remain, particularly in underrepresented populations due to the lack of comprehensive catalogues of genetic variation. Understanding these complex gene-gene interactions and the role of the environment underscores the importance of considering epistatic relationships when assessing MASLD risk and developing personalized therapeutic strategies, which could ease the future burden on healthcare systems.

## Key Points

· Metabolic dysfunction-associated steatotic liver disease (MASLD) affects nearly one-third of the global population, with rising incidence due to sedentary lifestyles, obesity, and type 2 diabetes.

· It is estimated that nearly 50% of MASLD patients have heritable predisposition genetic variants in genes such as *PNPLA3* and *TM6SF2*, which play significant roles in MASLD development.

· Several protective alleles have also been identified, which may mitigate MASLD progression despite genetic variants and environmental risk factors.

· Epistatic relationships between predisposing and protective genes can influence polygenic risk scores and improve risk stratification and early diagnosis of metabolic liver diseases.

· Risk-modifying variants exhibit different allele frequencies across populations.

## Introduction

Metabolic dysfunction-associated steatotic liver disease (MASLD), formerly known as NAFLD (Non-alcoholic fatty liver disease), is a worldwide problem on the rise. It is estimated that nearly a third of the world’s population suffers from this disease, and its incidence is projected to grow due to increasingly sedentary lifestyles, the obesity pandemic, and the rising prevalence of type 2 diabetes mellitus (T2DM) [1]. The annual healthcare cost per MASLD patient in the United States is projected to reach nearly 7,000 USD by 2039 [2], and the current annual economic burden resulting from this pandemic totals around 103 billion USD in the United States and about 35 billion EUR in the G4 European economic countries [3]. Given the large cost to public healthcare systems in recent years, and the need to improve patient quality of life, research efforts have focused on the prevention of liver diseases and the study of the different mechanisms that underlie MASLD.

In April 2023, the liver research community, supported by some of the most important hepatology associations in the world including the American Association for the Study of Liver Diseases (AASLD), the European Association for the Study of the Liver (EASL), and the Latin American Association for the Study of the Liver (ALEH), reached a consensus to change the nomenclature of non-alcoholic fatty liver disease (NAFLD) and non-alcoholic steatohepatitis (NASH) to “MASLD” (metabolic dysfunction-associated steatotic liver disease) and “MASH” (metabolic dysfunction-associated steatohepatitis), respectively. This new terminology focuses attention on the metabolic causes of steatotic liver disease, providing a more descriptive approach that better explains the pathogenesis of these conditions. In this review, the updated nomenclature is used throughout.

MASLD is an umbrella term that encompasses a continuum of liver pathologies. Simple steatosis, MASH, and liver fibrosis are considered the earliest stages of this spectrum and they are treatable and reversible [4]. Later stages include cirrhosis and hepatocellular carcinoma (HCC) which are considered irreversible and are associated with a poor prognosis and a higher death rate [5]. MASLD is a multifactorial disease closely related to a patient’s comorbidities (such as obesity and diabetes) and lifestyle risk factors (such as diet and sedentarism) [5–9]. A clearer understanding of the interplay between genetic and environmental risk factors and their overall impact on MASLD development and progression could help stratify risk and denote those patients where early decisive intervention could protect them against cirrhosis and HCC development.

Large-scale efforts have been undertaken to identify genetic predisposition factors, including genome-wide association studies (GWAS) and next-generation sequencing on large numbers of patients. It is estimated that between 25% and 75% of the heritability of MASLD is attributable to genetic factors [10]. As evidence of the increasing impact of genetics on different diseases, two-thirds of drugs approved by the Food and Drug Administration (FDA) have human genetic evidence [11]. These efforts have identified variants in genes such as *PNPLA3* (Patatin-Like Phospholipase Domain Containing 3) and *TM6SF2* (Transmembrane 6 superfamily member 2), which are now established as conferring prognostic information and are being used in the clinic for risk stratification, treatment selection and drug development [4].

In recent years, Allele Variants (AV) associated with mechanisms that seem to mitigate or delay the development of MASLD, sometimes even in the presence of acquired or environmental risk factors, have also been identified, henceforth referred to as “protective alleles”. For this review, AV are classified as either predisposing or protective based on their overall effect on MASLD development and progression. All AV discussed are among the most common in the European population according to gnomAD (v4.1.0). The reference allele is defined as the standard, and the alternative allele as the variant (GRCh38 is used as the reference genome assembly) [12]. 

Allele frequencies (AF) were obtained from gnomAD v4.1.0 across ten representative populations worldwide: Admixed American, African/African American, Amish, Ashkenazi Jewish, East Asian, European (Finnish), European (non-Finnish), Middle Eastern, and South Asian.

In the case of some genes such as *PNPLA3*, there is already a large amount of information available on its function and interactions, but in the case of other genes, their effect is emerging and there is still little information available. In this review, we have included those for which we believe that there is solid evidence as to their involvement in MASLD development and their interactions with other genes.

Intriguingly, evidence is accumulating that many alleles that modulate MASLD risk also modulate cardiovascular disease (CVD) risk, highlighting the importance of considering a systems approach when designing therapeutic interventions against MASLD risk factors. The interactions between these predisposing and protective alleles are complex and fascinating; here we aim to summarise the most current knowledge on their epistatic relationships.

### Genes associated with predisposition to MASLD

#### Patatin-like Phospholipase Domain Containing 3 (PNPLA3)

**Fig. 1:**
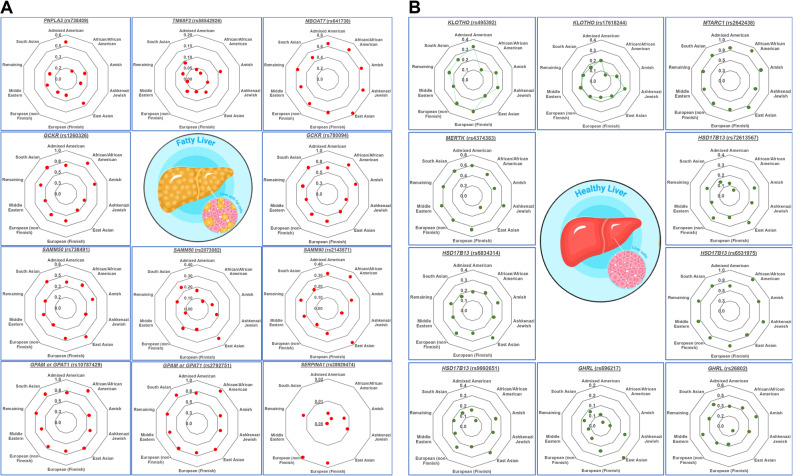
Genetic Ancestry Group Frequencies. Radar charts displaying allele frequencies of genetic variants associated with (**A**) MASLD predisposition and development and (**B**) MASLD alleles with protective effects across genetic ancestry groups. Each dot represents the allele frequency within a population, as categorised by gnomAD. Data were sourced from gnomAD v4.1.0, updated 9 October, 2024

The *PNPLA3* gene encodes for patatin-like phospholipase domain-containing protein 3 (also known as adiponutrin), a protein key to lipid metabolism [13]. The most widely studied variant is the *PNPLA3* rs738409 C > G [14, 15]. The Admixed American population has the highest AF value (0.5072), while the African/African American population has the lowest AF with a value of 0.1362 (Fig. 1A). This variant results in an isoleucine-to-methionine substitution at codon 148 (p.I148M) that reduces the hydrolase activity of the protein and compromises the retinyl ester release mechanism, leading to triacylglyceride accumulation inside liver cells [13, 16]. Fat accumulation in the liver is the first step in a cascade of effects that decreases hepatic resistance to fibrosis and inflammation; in fact, it is estimated that homozygosity for this allele increases the risk for developing MASLD-associated HCC by a factor of 8–10 depending on the population [14, 17].

*PNPLA3* rs738409 C > G also alters the retinol metabolism in the liver by decreasing the enzymatic activity of the retinyl-palmitate lipase, thereby decreasing retinol production and retinol release by hepatic stellate cells (HSCs) inducing their transdifferentiation into myofibroblast-like cells [18]. Furthermore, a recent study by Park and collaborators revealed that the expression of this allele (particularly in homozygous patients) also upregulated interleukin 6 (IL-6) signaling in these cells resulting in a proinflammatory state that favours the development and progression of MASLD [19].

There is evidence that this allele also has an effect on CVD. As intrahepatic lipid concentration increases, blood lipid concentration decreases. This shift in lipid allocation exerts a protective effect on cardiovascular system health. Luukkonen et al., found a direct association between the degree of insulin resistance and the degree of CVD protection conferred by this allele [20]. Patients with a score of four or higher for Homeostatic Model Assessment for Insulin Resistance (HOMA-IR), had lower concentrations of very low-density lipoprotein (VLDL) particles (which also presented lower diameters and lower triacylglyceride content) and low-density lipoprotein (LDL) particles (which presented with a lower cholesterol content), higher concentrations of HDL particles (with a larger diameter), and a lower apolipoprotein (apoB/apoA-1) ratio [20]. A similar relationship has been observed with obesity, meaning that patients at higher risk of developing severe liver disease have a higher protection against CVD [20].

Latin American populations display the highest frequencies of the G allele (according to gnomAD allele frequencies), with an overall frequency of 54.9% [17]. Examples of populations with elevated AF for this variant include Chileans (estimated to be 59%) [21] and Argentinians (calculated as 63.7%) [22]. However, estimates point to Mexicans having the highest *PNPLA3* rs738409 C > G allele frequency (77%) [23], which might contribute to the elevated risk of developing MASLD observed in this population [17, 21–23]. Given the increased risk of disease progression in carriers of this allele and the elevated allele frequency in the Mexican population, it is of no surprise that a 2023 study by Acuna et al. found that the Mexican-American population has the highest risk of HCC development in the United States and that this risk seems to increase in successive generations (37% in second generation and 66% in third generation of Mexican Americans) compounded by cultural assimilation (an increase in unhealthy habits) [24]. Opportune risk stratification, based on genotype and environmental markers in these populations could assist in early diagnosis and intervention to prevent disease progression and improve prognosis [25].

#### Transmembrane 6 Superfamily Member 2 (*TM6SF2*)

*TM6SF2* encodes for transmembrane 6 superfamily member 2 protein, which is involved in lipid and lipoprotein secretion by the liver [26, 27]. Expression of the rs58542926 C > T allele causes a glutamate-to-lysine substitution at codon 167 (p.E167K) which results in a reduction in hepatic VLDL synthesis and secretion. Consequently, given the role of VLDL as one of the most important transporters of triacylglycerol and cholesterol from the liver to peripheral tissues, mainly the adipose tissue, this allele causes lipid accumulation within the liver resulting in hepatic steatosis (assessed through hepatic ultrasound) [26, 28, 29]. 

This effect, while deleterious to the liver, decreases total cholesterol levels in the bloodstream, thereby preventing dyslipidemia and the inherent CVD risk associated with it [26, 28, 29], also induces a reduction of APOB secretion by a possible instability in the protein validated in human hepatic spheroids [30]. In fact, Lee et al. report that paediatric Korean patients carrying this allele showed a correlation with significantly lower levels of serum total cholesterol (TC) and triacylglycerides (TG) (genotype TC, *p* = 0.033/TT, *p* = 0.338) [31]. Overall, this single-nucleotide polymorphism (SNP) is associated with increased MASLD progression and hepatocellular damage as evidenced by elevated serum levels of alanine aminotransferase (ALT), aspartate aminotransferase (AST) and total bilirubin [32], but lower CVD risk [31]. The Amish population presents the highest AF for this gene with a value of 0.1143, and the African/African American population has the lowest value with 0.0327 (Fig. 1A).

 Collectively, these findings suggest that this variant may confer cardiometabolic benefits in certain populations, despite its established association with liver disease severity.

#### Membrane-Bound O-Acyltransferase Domain Containing 7 (MBOAT7)

This gene is mostly expressed by hepatocytes, HSCs, and sinusoidal cells and encodes an acyl-transferase enzyme, also known as lysophospholipid acyltransferase 7. This enzyme catalyzes the reacylation of phospholipids as part of the Lands cycle, a key step in phospholipid metabolism that enables the selective placement of fatty acyls chains. The *MBOAT7* rs641738 C>T variant leads to gene downregulation, resulting in reduced protein expression and has been linked to increased hepatic triacylglycerol synthesis [14, 17, 33, 34]. This allele is significantly associated with advanced fibrosis in both MASLD (odds ratio [OR] 1.22) and alcoholic liver disease in patients of European descent [35]; there is still conflicting evidence on its role in HCC development in both diseases [35, 36]. As shown in Fig. 1A, the East Asian population presents an AF value of 0.7739, the highest among these ten populations, while the South Asian population presents the lowest frequency with a value of 0.4767. A study by Xu et al. in a cohort of 1287 elderly Chinese individuals associated this allele with a reduced CVD risk (OR = 0.570) [37]. Similarly, a meta-analysis by Teo et al. associated the presence of this allele in populations of European descent with lower serum triacylglycerides and cholesterol levels [35].

#### Glucokinase Regulator (GCKR)

Variants in *GCKR* have also been associated with MASLD. This gene is predominantly expressed in the liver and encodes the glucokinase regulatory protein (GKRP), which regulates hepatic glucokinase. Glucokinase is active in both liver and pancreatic tissues, where it plays a key role in glucose homeostasis. Glucose uptake by the liver has a positive correlation to de novo lipogenesis by providing the necessary substrate for glycerol moiety and acetyl molecules for hepatic lipid synthesis. The *GCKR* rs1260326 C > T allele encodes for a proline-to-leucine substitution at codon 446 (p.P446L) of the protein, which is unable to downregulate glucokinase activity causing an increased influx of glucose into the liver which promotes the development of hepatic steatosis [14, 38, 39]. Paradoxically, as hepatic glucose storage increases, circulating fasting glucose and insulin levels throughout the body decrease, which in turn lowers the risk of developing T2DM. This allele has also been associated to increased CVD risk and increased serum TG and TC (by way of increased LDL levels and despite low HDL levels); this effect increases in patients with T2DM due to a further increase in *GCKR* activity [40]. Additionally, the rs780094 G > A allele has also been linked to MASLD development and progression but there is scant information on the exact mechanisms of action of this allele [14, 38, 39]. For both alleles, rs1260326 C > T and rs780094 G > A, the African and African American population has the highest AF value (0.8608 and 0.8254 respectively), and the Ashkenazi Jewish population has the lowest frequency with an AF value (0.4662 and 0.4565 respectively) (Fig. 1A).

#### Sorting and Assembly Machinery Component (SAMM50)

In recent years, polymorphisms in the *SAMM50* gene have been identified in Asian and Hispanic populations as risk factors for the development and increase in severity of MASLD [31, 41]. This gene codes for the *SAMM50* protein, an outer mitochondrial membrane protein responsible for maintaining organelle integrity and morphology, regulating both mitophagy and the removal of reactive oxygen species [41]. Two polymorphisms have been associated with this disease, rs738491 C > T and rs2073082 A > G, both affecting mitochondrial function and decreasing the expression of genes related to fatty acid oxidation and, by extension, ketone body production. As a result of this loss of function, hepatocytes increase intracellular lipid accumulation; additionally, higher levels of serum ALT, AST and TG were observed in patients carrying these variants [31, 41]. The *SAMM50* rs2143571 G > A allele has also been associated with MASLD development in Chinese, Japanese, Indian, and Mexican populations [42]. The East Asian population presents the highest AF values for the three *SAMM50* variants (rs738491 C > T = 0.4888, rs2073082 A > G = 0.3323, and rs2143571 G > A = 0.3876) (Fig. 1A).

#### Glycerol-3-phosphate acyltransferase (GPAM)

This gene encodes for the glycerol-3-phosphate acyltransferase protein, a mitochondrial enzyme that catalyses the first step (a rate-limiting step) in the de novo glycerophospholipid synthesis pathway [43, 44]. This protein is most often located in the outer membrane of the mitochondria in hepatocytes, although Yu et al. (2018) reported relatively high levels of it in adipose tissue [43]. Its overexpression has been observed to cause hepatic steatosis, damage, and peripheral and hepatic insulin resistance [43–45]. This enzyme is known to steer fatty acid metabolism toward glycerophospholipid synthesis and away from beta-oxidation [44]. Two alleles for this gene are linked to MASLD in the literature: rs10787429 T > C and rs2792751 T > C.

Expression of the *GPAM* rs10787429 T > C allele, with a gain of function mutation, is associated with increases in total cholesterol (by way of increased LDL and HDL cholesterol), ALT, AST, alkaline phosphatase (ALP), and total bilirubin levels. It is usually co-expressed with genes linked to VLDL formation. This allele increases the risk of developing Alcoholic Liver Disease (ALD, using the original authors’ nomenclature) (OR 1.34) and MASLD (OR 1.18), in an allele dose-dependent manner [44]. The global AF for this particular allele (0.7654) is higher than those observed in previous SNPs; frequency values for individual populations are similarly high, the highest being that of the African/African American population with a 0.9150 value (Fig. 1A). The *GPAM* rs2792751 T > C allele, a missense variation, has also been found associated with high LDL, HDL, and TC levels, and higher triacylglyceride content in hepatocytes [46]. This allele presents a global AF of 0.7326, with the highest value present in the Africa/African American population (0.9314) and the lowest (0.5714) in the Amish Population.

 Recently, a MASLD/NASH risk-conferring effect has been reported for a couple of *GPAM* variants. Hakim et al., 2025 have reported two novel *GPAM* loci from analysis from the UK Biobank, located at rs55875049, which showed strong protection against fibrosis and steatohepatitis in adults but not in children with MASLD, and at rs10787429, which showed protection against fibrosis in children but not in adults [47]. A better understanding of how these variants interact, would bring us closer to precision medicine, which would consider the relationship between genetic variants and age.

#### Autophagy Related 7 Homolog (ATG7)

Autophagy Related 7 Homolog (*ATG7*) is a key protein involved in autophagy, particularly in recycling damaged mitochondria and mediating the transport of cytoplasmic vesicles. Dysfunction of ATG7 impairs autophagy, leading to hepatic lipid accumulation and inflammation, thereby promoting the progression of MASLD [48]. Rare and low-frequency variants of *ATG7* have been associated with more severe MASLD phenotypes, including advanced fibrosis and hepatocellular carcinoma. Notably, the rs143545741 C > T (P426L) and rs36117895 T > C (V471A) variants result in loss of function [48]. In mouse models, hepatocyte-specific *Atg7* deletion induces liver enlargement, fibrosis, and tumorigenesis [49]. In human samples, increased hepatic *ATG7* mRNA and protein expression have been observed in MASH, particularly in association with obesity, and correlated with markers of inflammation when compared to simple steatosis [50]. Although both alleles present very low frequency, the Amish population shows the highest reported frequencies, with rs143545741 and rs36117895 having AF of 0.01206 and 0.1140, respectively.

#### Serpin Family A Member 1 (SERPINA1)

*SERPINA1* is a gene that encodes Alpha 1-antitrypsin (A1AT), a protein that inhibits serine proteases such as the neutrophil elastase thereby preventing lung and liver damage [51]. When the *SERPINA1* rs28929474 C > T allele is present in a homozygous state, it leads to A1AT deficiency (A1ATD), a relatively common genetic disease that causes liver disease and chronic obstructive pulmonary disease [51]. Although the mechanisms for liver disease development under A1ATD are different from those in MASLD [51, 52], recent studies have shown that one copy of the allele can increase the risk of chronic liver disease with an odds ratio of 1.64, which is similar to the risk associated with the *PNPLA3* rs738409 A > G allele (1.48) [52, 53]. In addition, the effects of this allele on a patient’s burden for liver disease become more pronounced with age [53]. Research on European populations has revealed a higher prevalence of this allele among patients with MASLD or ALD compared to healthy individuals [54, 55]. This allele is mostly found in the European (Non-Finnish) population, with an allele frequency of 0.01939, closely followed by the European (Finnish) population with an AF of 0.0185.

Up to this date, *PNPLA3*,* TM6SF2*,* MBOAT7*,* GCKR*, *SAMM50*, and *GPAM* remain as canonical genes associated with MASLD predisposition and/or development and present the largest number of studied allele variants. It may come as no surprise that given recent technological advancements and the plummeting costs of sequencing technologies, additional genes hypothesized to play a role in the development of MASLD have been identified. However, there is still a need for more research to strengthen the evidence for these assertions.

### Genes associated with a protective effect in MASLD

#### Cell death-inducing DFFA Like Effector B (CIDEB)

 Cell death-inducing DFFA Like Effector B (*CIDEB*), a member of the CIDE protein family, is a liver-specific protein associated with lipid droplet formation and aggregation. *CIDEB* participates in the formation and maturation of triacylglycerol-enriched VLDL particles in hepatocytes [56]. Also, this gene is involved in apoptotic and oxidation processes, as well as insulin sensitivity [57, 58].

A whole-body *Cideb* knockout mouse model fed with a high-fat diet (58% fat) showed lower levels of plasma triacylglycerides and free fatty acids than the wild-type group. In addition, these mice were resistant to high-fat diet-induced obesity and liver steatosis [57]. This study illustrates the importance of this gene in mediating metabolic processes related to lipid metabolism, and its contribution to conditions such as obesity, insulin resistance, and hepatic steatosis.

Moreover, Verweij et al. (2022) reported a 53% reduction in the risk of MASLD development in patients carrying rare loss-of-function mutations in one of two copies of the *CIDEB* gene and a 54% reduction in the risk of developing MASLD-related cirrhosis [59]. They also identified *CIDEB* genetic variants that confer powerful protection to the carriers by decreasing alanine aminotransferase serum levels. Even though these variants have not been widely described, there are efforts to develop drugs that mimic this effect [57, 59].

#### Klotho (KL)

The Klotho protein family (comprised of four homologous proteins: alpha, beta, gamma, and KLrP) has been associated with pleiotropic effects related to the regulation of phosphate homeostasis, reduction of oxidative stress, and suppression of inflammation, and also serves as a co-receptor of endocrine fibroblast growth factors [60–62]. Of these four proteins, alpha and beta klotho have been associated with protective effects against metabolic dysfunctions and fatty liver disease development.

The *𝛼-Klotho (𝛼-KL)* rs495392 C > A allele has been recently associated with a protective effect against hepatic steatosis reducing the severity of the disease [63]. Although this allele variant occurs in an intronic area and therefore does not induce a protein change, it affects *KL* gene expression, altering lipid accumulation in the liver [63]. Research on the mechanisms responsible for this effect is still ongoing; however, vitamin D levels have a crucial role in the protective effects of this allele [63–65]. The Admixed American population has the highest AF value for the *𝛼-KL* rs495392 C > A allele (0.3302), while the African/American population presents the lowest frequency value (0.1226) (Fig. 1B).

The protective role of β-Klotho (β-*KL*) is recognized through co-receptor activity with the fibroblast growth factor receptor 4 (FGFR4), and was discovered serendipitously. Under a wild-type condition, it induces protective effects against lipotoxicity and inflammation in hepatocytes [65, 66]. However, patients with MASLD carrying the rs17618244 variant in the *KLB* gene showed decreased serum levels of β-Klotho protein, which was associated with an increased risk of hepatic fibrosis, inflammation, and cirrhosis [65, 67].

#### Mitochondrial Amidoxime Reducing Component 1 (MTARC1)

The role of the *MTARC1* gene has been widely associated with detoxification reactions, oxidoreductase activity (as in the reduction of nitrite for the production of nitric oxide), activation of N-hydroxylated prodrugs, and other metabolic processes [68, 69]. Interestingly, recent studies have reported novel missense variants in *MTARC1*, including rs2642438 G > A and a rare protein-truncating variant, rs139321832 C > G/C > T, that confer protection against the development of MASLD [68, 70–72]. The occurrence of the *MTARC1* rs2642438 allele (A165T) is higher than that of the rs139321832 allele (p.R200Ter), which produces a truncated protein. *MTARC1* rs139321832 C > G has a reported AF in gnomAD of 0.000011 in the South Asian population, while for the C > T variant, the Ashkenazi Jewish population has the highest AF at only 0.0042 (Fig. 1B). The protective mechanism by which these alleles act remains elusive [70].

In a study from 2020, Emdin et al. analysed genotyping and blood sample data from 12,361 all-cause cirrhotic patients and 790,095 controls from eight different cohorts. Their results suggested that carriers of the rs2642438 G > A variant presented lower hepatic fat, and lower serum levels of alanine transaminase and alkaline phosphatase [70]. Additionally, the authors also report that these patients presented decreased serum levels of LDL and TC and that this allele was not associated with increased CVD risk [70]. Moreover, European, African American, and Black British carriers showed similar test results [68]. The risk of developing MASLD was ∼15% lower in homozygous and/or heterozygous allele carriers than in non-carriers (GA/AA vs. GG), and the risk of dying from liver disease was 39% lower in homozygous carriers. Furthermore, the protective effect of this variant was amplified in patients with obesity, T2DM, and in the presence of the *PNPLA3* rs738409 C > G allele by up to 50% [68].

Interestingly, a GWAS and histologic analysis of 1,450 children suggested a lower grade of hepatic steatosis in those children carrying the rs2642438 G > A variant [71]. The data did not show any change in the expression of *MTARC1* in the liver or an impact on lipid metabolism in the presence of this variant. However, the authors observed a destabilization and reduction in the metal-binding capacity of the protein [71]. Further studies also confirmed the association between *MTARC1* and MASLD progression in paediatric patients.

There is a lack of comprehensive data on the *MTARC1* rs139321832 AF values; however, for the rs2642438 G > A allele that the African/African American population presents the highest AF values (0.9196) when compared to the other seven populations (Fig. 1B).

#### Myeloid-Epithelial-Reproductive Tyrosine Kinase (MERTK)

The *MERTK* gene encodes for a cell surface protein receptor of the Tyro3-Axl-MerTK receptor tyrosine kinase family (TAM), mainly involved in cell survival, migration, differentiation, and efferocytosis (phagocytosis of apoptotic cells) [10]. Additionally, *MERTK* has an important role in the immune response, apoptosis, HSC activation (which triggers profibrotic processes), and steatosis modulation [73–75]. The *MERTK* rs4374383 G > A allele can potentially protect against fibrotic processes by reducing its protein expression [73, 76]. The allelic frequency is reported to be higher in the European (Finnish) population (0.6696), followed by Ashkenazi Jewish (0.6526), Middle Eastern (0.6294), and European (non-Finnish) populations with 0.6111 followed by other populations with values as low as 0.27. (Fig. 1B).

Petta and collaborators analyzed data from two Italian cohorts; one consisting of 533 patients with suspected MASH but without severe obesity, and another comprising 158 patients without any metabolic disturbance and with normal liver enzyme levels. Their findings suggest a protective effect induced by the *MERTK* rs4374383 G > A variant against fibrosis at F2-F4 stages in homozygous patients diagnosed with MASLD. The mechanism of action was associated with a modulation of HSC activation given a decrease in the intrahepatic activation of *MERTK* [73]. A lower incidence of MASLD and T2DM was evidenced in variant carriers, even in those with Hepatitis C infection, which presented a milder form of the disease [74, 75].

The *MERTK* receptor induces the ERK-TGFβ1 pathway, which activates HSCs, triggering the development of liver fibrosis [77]. It has been reported that mice treated with RU-301, an inhibitor of the functional interaction between GAS6 and MERTK, showed a significant reduction in MASH-induced fibrosis, attenuation of ERK activation, and lower TGFβ1 expression [78]. These findings highlight the role of *MERTK–TGFβ1* signaling in the macrophage-HSC crosstalk in MASLD progression.

#### Hydroxysteroid 17-beta dehydrogenase 13 (HSD17B13)

The *HSD17B13* gene encodes for hydroxysteroid 17-beta dehydrogenase 13, a redox enzyme, and it is mainly expressed in hepatocytes although it can also be found in other hepatic cells. This protein promotes lipogenesis and has an important role in liver lipid droplet regulation. This gene, under adverse environmental conditions (high fat diet, sedentarism, etc.) may contribute to increasing the risk of developing MASLD; however, some of its variants can protect from the pathogenesis of this disease [79].

The *HSD17B13* rs72613567 T > A allele, which induces the production of a truncated protein with loss of function, has been associated with lower levels of circulating transaminases and a lower risk for developing MASH and liver fibrosis [79–81]. The risk of HCC was also reduced in heterozygous (OR = 0.65) and homozygous (OR = 0.289) patients [79]. Furthermore, Emdin et al. demonstrated that this allele is not associated with an increased risk of CVD and therefore could become a potential therapeutic target [70].

 Other variants of this gene with protective effects include the rs6834314 A>G allele, which is associated with lower scores of liver inflammation [82]; the rs6531975 G>A allele, which protects against hepatic fibrosis [83]; and the rs9992651 G>A allele, found in a non-coding region of *HSD17B13*, which also protects against MASLD [82].

All eight populations showed low AF values for the *HSD17B13* rs72613567 T > A, rs6834314 A > G, and rs9992651 G > A alleles. However, for the rs6531975 G > A allele, the Admixed American population presented the lowest AF value (0.6186), while the African/African American population presented the highest AF value (0.9445) (Fig. 1B).

#### Ghrelin (GHRL)

Although the Ghrelin gene (*GHRL*) is expressed mainly in the gastric parietal cells P/D1 (epithelial gastric cells that secrete hydrochloric acid), its relationship to MASLD development has been thoroughly established [84, 85]. This gene plays a crucial role in the balance between food intake and appetite, as well as in the metabolic regulation of glucose and lipids, all common processes in both obesity and MASLD [84, 85].

Lower circulating levels of ghrelin have been associated with an increase in the incidence of MASLD by way of an increase in food intake and therefore obesity, insulin resistance and T2DM [85]. To illustrate these effects, Tabaeian et al. demonstrated that the exogenous administration of ghrelin decreases liver injury, oxidative stress, and hepatic inflammation. This suggests that higher levels of ghrelin may be used to decrease obesity and insulin resistance, and protect against MASLD [85]. The *GHRL* rs696217 G > T variant induces a higher hormone serum rate and it is associated with a lower body mass index (BMI), triacylglyceride, and cholesterol levels, and a milder form of T2DM [85]. Another SNP that has been studied in this gene is the rs26802 T > G allele, an intronic variant that has been associated with a decrease in MASLD susceptibility, although its mechanisms are yet to be characterized [84].

The East Asian population presents the highest AF for the rs696217 G > T with a value of 0.1934, the other seven populations present similarly low AF values. In contrast, the East Asian population presents the lowest AF value for the rs26802 T>G variant (0.0837), while the Amish and Middle Eastern populations have the highest AF values (0.4251 and 0.4108, respectively) (Fig. 1B).

### Epistatic relationship between MASLD modulators

Epistasis refers to the phenomenon in which the expression and function of certain genes are modified due to the expression of other genes, and which can sometimes influence the phenotype in health or pathological conditions. It is intriguing to consider the mechanisms through which these synergistic or antagonistic interactions might take place. The effect of epistatic relationships in MASLD development has just started being explored. In this section, we will discuss the genetic interactions that can potentiate or inhibit the evolution of this disease.

In order to understand the impact and utility of precision medicine, and also the elucidation of epistatic relationship among genetic variants, Troppmair et al. (2025) have shown that epistatic relationship between gene variants present in donor and recipient is important for liver transplant. While the usual criteria used for liver transplant are some variables as age, absence of maladies and immunocompatibility, Troppmair et al., conducted a retrospective study of 435 liver transplant recipients evaluating the diversity of *PNPLA3*, *TM6SF2*, and *HSD17B13* genotypes in both liver donors and transplanted recipients. The main findings revealed that approximately 34% of transplanted patients developed steatosis, with a median onset of 8 years post-transplantation, despite having been evaluated as healthy prior to the transplant [86].

Additionally, the presence of the *TM6SF2* rs58542926 C > T variant in the transplanted liver was associated with reduced survival in liver transplant recipients, with median survival decreasing from 16 years to 9 years, even if the recipient does not present the variant [86]. These novel findings will be useful for increasing survival rate in patients with liver transplant, but at the same time, it reinforces the impact of some gene variants as part of personalized medicine in liver transplant.

The *PNPLA3* rs738409 C > G allele is the most commonly studied MASLD genetic risk factor. Concurrent expressions of *PNPLA3* rs738409 C > G and *TM6SF2* rs58542926 C > T alleles have been linked with an increased risk of MASH and fibrosis development when compared to their individual expression [87, 88]. Xu et al. demonstrated this synergistic effect describing a 5-fold increase in MASLD prevalence in subjects presenting both variants compared to non-carriers or those presenting only one variant [89]. Given that the *PNPLA3* risk variant can cause both steatosis and fibrosis, we hypothesise that the addition of *TM6SF2* risk allele potentiates the steatosis effect through its activities in synergistic pathways. A faulty *TM6SF2 *protein may cause an accumulation of lipids within hepatocytes, which could be taken in by the deficient adiponutrin and enhance the long-term storage of lipid droplets in the liver.

Furthermore, in 2022, Longo and collaborators focused on the effects of the combination of the *PNPLA3* rs738409 C > G, *TM6SF2* rs58542926 C > T, and *MBOAT7* rs641738 C > T alleles; their results show that carriers of these three alleles present a more aggressive MASLD phenotype that tends to progress rapidly towards HCC. Indeed, the patients that presented the combination of these three variants had a two-fold increase in risk of developing HCC [90]. This combination seems to affect lipid composition and metabolism, and favour organelle derangement (particularly mitochondrial derangement) that results in a cellular pro-survival phenotype that tends towards metabolic reprogramming.

Interestingly, the protective effect of *HSD17B13* rs72613567 T > A allele is not observed when expressed in conjunction with the *PNPLA3* rs738409 C > G and *TM6SF2* rs58542926 C > T alleles. The mechanics of this loss of effect are not fully understood, questions remain on whether the protective effect of this allele is surpassed by the combined effect of the other two or whether it becomes a risk allele in itself. Gellert-Kristensen et al. (2022), found that the presence of these three alleles caused a 12-fold increase in the risk of presenting cirrhosis and a 29-fold increase in the risk of presenting HCC when compared to the general population [91]. However, a study focusing only on the interaction between *HSD17B13* rs72613567 T > A and *PNPLA3* rs738409 C > G alleles found that the protective effect against liver injury of the *HSD17B13* variant persisted. Lower circulating ALT and AST levels, as well as decreased expression of *PNPLA3* mRNA were observed in carriers of both alleles (dose-dependent effect) [80].

In 2012, Santoro and collaborators found that the co-expression of *PNPLA3* rs738409 C > G and the *GCKR* rs1260326 C > T alleles increased the hepatic fat content in obese children and adolescents from 15 to 39%, according to their ethnicity [92]. No other studies regarding this relationship were found during our revision of the literature; thus, it represents an area of opportunity for future research.

Additionally, a synergistic effect between the *PNPLA3* rs738409 C > G and the *SAMM50* rs738491 C > T alleles has been described in conjunction with a third variant depending on the study in question. Kitamoto et al. and Xu et al. (2013) found that these polymorphisms in conjunction with *parvin beta (PARVB)* rs5764455 G > A were observed in MASLD Chinese and Japanese patients with lower triacylglyceride levels and higher transaminase levels when compared to a healthy control population. These changes were found to have a synergistic effect that increases the predisposition to developing MASH [93, 94]. Similar results were found by Lee et al. (2022), where the *PNPLA3* and *SAMM50* risk variants interacted with the *TM6SF2* rs58542926 C > T allele to increase circulating transaminase and alkaline phosphatase levels as well as the overall MASLD severity as measured by the FIB-4 score in paediatric patients [31].

In contrast, there are epistatic interactions between *PNPLA3* and previously mentioned protective alleles that can reduce its deleterious effect. Information on the interaction between these variants is scarce. Liu et al. (2022), studied the effects of the co-expression of the *KL* rs495392 C > A and the *PNPLA3* rs738409 C > G alleles in a Chinese cohort. They found that patients with both alleles presented a milder form of MASH than carriers of the *PNPLA3* rs738409 C > G allele alone. These observations were further validated in 2,798 European participants from the Rotterdam Study cohort [63].

A similar protective effect was observed in the interaction between *PNPLA3* rs738409 C > G allele and *MTARC1* rs2642438 G > A alleles. Schneider and collaborators (2021) identified through a genomic analysis in UK Biobank participants that the effect of the *MTARC1* variant was amplified in patients with obesity, T2DM, and the presence of *PNPLA3* allele. Additionally, *MTARC1* rs2642438 G > A variant carriers presented a 50% decrease in liver-related mortality when having T2DM or expressing the *PNPLA3* risk allele in comparison to patients that presented this variant alone [68].

Finally, the *HSD17B13* rs72613567 T > A allele was also associated with a decrease in liver damage in adult patients carrying the *PNPLA3* rs738409 C > G allele [79, 80]. In obese paediatric patients, it induced a reduction in liver damage, regardless of the presence of risk variants in *PNPLA3*, *TM6SF2*, and *MBOAT7* [67, 90]. The *HSD17B13* rs6531975 G > A allele has been found to decrease the effects of *PNPLA3* rs738409 C > G allele in patients with hepatic steatosis [83].

**Fig. 2:**
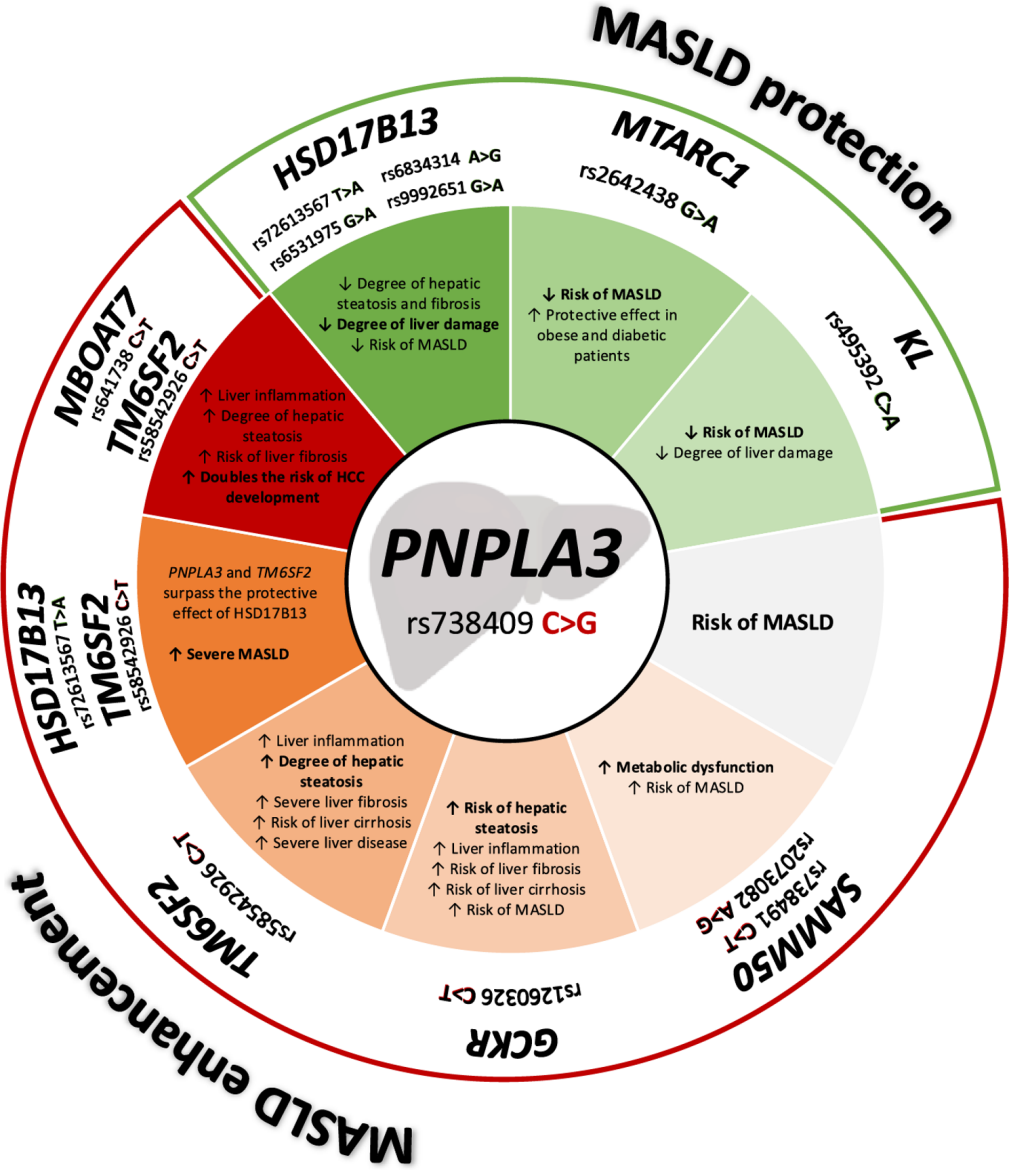
Epistatic interactions of *PNPLA3* rs738409 C>G allele with gene variants associated with MASLD predisposition and protection. The *PNPLA3* rs738409 C>G allele independently increases the risk of developing MASLD. However, its co-expression with variants of *SAMM50*, *GCKR*, *TM6SF2*, and *MBOAT* genes can exacerbate disease severity. Conversely, variants in *HSD17B13*, *MTARC1*, and* KL* genes exert protective effects, mitigating MASLD risk. The colour gradients represent the extent of damage or protection conferred by each genetic interaction. Darker color shades indicate stronger effects

To the best of our knowledge, this is the first approach to reviewing the epistatic relationships between known risk and protective alleles present in MASLD development (Fig. 2). There are still large gaps in our knowledge related to this topic; therefore, further research including interdisciplinary approaches and diverse research subjects should hopefully lead to the development of new forms of diagnosis and treatment of this disease. 

### Beyond in vivo models: modern approaches to study MASLD complexity

MASLD studies represent a huge challenge, based on the complex interaction of the different hepatic cell types and the multiple processes that take place simultaneously in the liver. The biological variability of patients and a multifactorial environment further complicate this convoluted landscape. To study all these factors, in vitro, in vivo, and in silico models are evolving and constantly being improved to allow simultaneous control of multiple variables, and a more accurate approximation of human biological and clinical reality.

The recent emergence of more versatile systems and more powerful computational tools has benefited the practice of translational medicine. In this context, microphysiological systems (MPS) have allowed the study of MASLD to go beyond the conventional 2D cell culture to the implementation of a more natural, physiologically relevant environment. MPSs have become consolidated 3D microfluidic platforms with the potential to combine multiple cell types to create systems with high similarity to the overall organ structure, and the capability to provide cell-to-cell communication using several cellular types such as human primary cells, immortalized cell lines, and induced pluripotent stem cells (iPSCs) (Table 1) [95].

MPS have rapidly evolved from organoids to Organ-on-a-Chip technology including micropatterned tissue constructs, resulting in systems that replicate the native tissue physiology (Table 1). Several reports have applied the use of MPS platforms to improve our understanding of hepatic alterations during fatty liver and liver fibrosis with interesting mechanistic results [96, 97]; however, MPSs are yet to be used to characterize the genetic and epistatic interactions that sustain the onset and development of MASLD.

Artificial Intelligence systems underlie the Machine Learning and Deep Neural Network methods. These techniques can learn patterns from data and make predictions based on previous experiences to make the recognition of complex patterns feasible [98]. Recently, this approach has been used to understand the relationship of MASLD complexity with cardiac disabilities [99] and explore possibilities of therapeutic strategies in liver care [100].

Overall, the mimicry of physio-pathological conditions with approaches based on new cellular and biophysical strategies, as well as advanced computational skills, make possible the implementation of versatile precision medicine platforms in a near future.

**Table 1:** Novel models to study MASLD and other liver pathologies to improve the understanding of epistatic relationships. Different techniques are presented, focusing on mimicking clinical characteristics, thereby enabling a better study of the epistatic interactions of different genetic variants. These novel techniques may help promote the development of personalized treatments to improve life expectancy

Technique	Definition	Studies
**Induced pluripotent stem cells (iPSCs)**	As a preclinical model, induced pluripotent stem cells (iPSCs) can be generated from adult somatic cells, such as those from the skin or blood, by reprogramming them into an embryonic-like state through the expression of specific genes. A simple search in the National Center for Biotechnology Information (NCBI) database using the terms “iPSC” and “MASLD”, yielded only nine related studies, highlighting how recent and emerging this approach is in the context of MASLD. We present the studies with the greatest relevance and impact in this area.	A) Can predict the risk of lipid accumulation on in vitro conditions using iPSC-based MASLD cells. Treatment with Oleate induced higher intracellular lipid accumulation in subject-derived iPSCs from patients with MASLD or MASH compared with the control group [101]. B) Through gene editing in human-induced pluripotent stem cells, a model to mimic the effects of the *TM6SF2*-E167K mutation was developed. Hepatocytes carrying the variant recapitulated, under in vitro conditions, the detrimental effects observed in hepatocytes from human carriers of the variant [102]. C) In a biomimetic preprint by Xia et al. (2024), a preclinical evaluation of resmetirom effects in iPSC was implemented. Using cells from *PNPLA3* wild type patients, or homozygotes and heterozygotes of the *PNPLA3* rs738409 (I148M) variant, they created “patient biomimetic twins” (each assay was replicated with cells from the same patient with or without the drug). Interestingly, the model reproduced in vitro the main signatures of MASLD in patients carrying the variant (increased steatosis, stellate cell activation and secretion of pro-fibrotic markers in *PNPLA3* GG carriers) [103]. This model has opened the door to a better understanding of the effects of this variant, and to improved treatments, especially for Latin American populations, in whom the variant is present in over 50% of individuals [23].
**Chips**	Microfluidic chips have been used to evaluate the inter-communication between different type of cells from the same organ or different organs without physical contact, only a communication through very small channels covered with fluids.	A) Effects of the drugs lanifibranor and resmetirom were tested using multicellular biochip-based liver sinusoid model. Mouse primary hepatocytes, hepatic stellate cells, kupffer cells, and endothelial cells were seeded in a dual-chamber biocompatible liver-on-a-chip (LoC). This setup maintains the communication between these types of cells while allowing control over “who speak first or after”, enabling to identify the contribution of each cell type to MASLD development and how the drugs modify each important contribution. As a result, lanifibranor prevents the onset of inflammation, while resmetirom decreases lipid accumulation in hepatocytes [104]. B) The liver acinus microphysiology system (LAMPS) is an experimental model constructed with patient-derived primary cells, designed to be biologically accurate (biomimetic) by incorporating the three-dimensional layered structure of the liver acinus. By creating a microenvironment with gradients of nutrients and oxygen, these systems can establish a physiological metabolic zonation similar to that in a living liver. Xia et al. replicated steatosis, fibrosis, and immune activation in LAMPS derived from patients carrying the *PNPLA3* I148M variant, and through resmetirom treatment achieved efficacy in the *PNPLA3* wild-type CC LAMPS compared to the GG variant across multiple MASLD metrics [105]. C) Mimicking the architecture and functional parameters of liver in a little chip is possible by Organoid-on-a-chip (OoC) which involves a microfluidic chip integrating multi-channel 3D cell cultures. This model enhances the understanding of MASLD by recreating the different stages and allows for multi-drug screening [106].
**Spheroids**	Spheroids are three-dimensional (3D) cell culture models that self-assemble into spherical structures and are widely used to mimic tissues and microtumors in vitro. Compared with conventional 2D cultures, spheroids better reproduce the in vivo microenvironment by supporting cells in different functional states, such as proliferative and hypoxic conditions. Therefore, they are valuable tools in regenerative medicine and drug testing.	A) Evaluation of hypoxia comparing internal cells of spheroids and peripheral cells revealed the expression and stabilization of Hypoxia-inducible factor 1alpha (HIF-1alpha), which promotes tumour cell survival and progression under hypoxia in HCC cell models [107]. B) A subset of genes (*GAS7, SPON1, SERPINE1, LTBP2, KLF9, EFEMP1*) driving MASH-related activation of HSCs was identified through dicer substrate interfering (dsi) RNA-based gene knockdown. These genes were evaluated in 2D and 3D spheroids culture, with *SERPINE1* being the main gene protecting mice from MASH-related liver fibrosis [108].
**Organoids**	Liver organoids are 3D in vitro structures that mimic the function and architecture of liver using two or more liver cell types. One of the main challenges is identifying the optimal cell ratio to reproduce liver processes as closely as possible. For liver studies, the most common combination of cells is hepatocytes, THP-1-derived macrophages, and HSCs.	A) Three conditions to induce MASLD in vitro were evaluated using oleic acid, palmitic acid or TGF-β1 stimulation. After treatment, single-cell transcriptomic analysis showed that oleic acid reduced the stellate cell population and decreased fibrosis deposits. In contrast, palmitic acid and TGF-β1 produced a pattern that more closely resembled MASLD in humans, with inflammation and fibrosis development [109]. B) Liver zonation is a challenging characteristic to replicate in vitro but represents a key feature of the basic liver unit study, the lobule. Reza et al. (2025) developed a self-assembling zone-specific liver organoid through co-culturing ascorbate- and bilirubin-enriched hepatic progenitors derived from human induced pluripotent stem cells. This created, for the first time, in vitro multizonal organoids with consistently urea cycle, glutathione synthesis, and glutamate synthesis zonation [110]. C) Through CRISPR screening in organoids of human fetal hepatocyte organoids, the *FADS2* gene (fatty acid desaturase 2) has been discovered as a likely key determinant of hepatic steatosis. This gene has the capacity to reduce de novo lipogenesis, thereby decreasing lipid storage in the organoid cells [111]. D) An exciting breakthrough in organoid modeling has been made recently by Abilez et al., 2025. Abilez and colleagues have managed to generate organoids that develop vascularization. This represents a milestone, since they successfully generated organoids that allow a more realistic evaluation of MASLD, as these ensure homogenous exposure to stimuli such as drug treatment or other therapies during organ development [112].
**Spatial tissue study**	Although single-cell studies have provided relevant information about the genomic and transcriptomic profiles in specific healthy or disease conditions, spatial biology has emerged as a more integral and complete model. The ability to asses different cell types in their position in their specific environment has enhanced the understanding of the contribution that each cell makes, while simultaneously considering its role within the tissue context [113].	A) According to Balanchander et al. (2025)“existent liver models present low reproducibility, incomplete disease features, and poor correlation with clinical drug responses” [114]. They have developed a new platform called liver-on-a-chip for NASH drug testing (LEADS). This model recapitulates the pathological stages of MASH, scoring disease severity through microscope evaluation and secretome analysis. They were able to reproduce clinical observations in MASH patients treated with resmetirom, saroglitazar, pioglitazone, cenicriviroc and obeticholic acid. The basis of this technique is a microfluidic chip platform with co-culturing human adult liver stem cell (haLSC)-derived hepatobiliary organoids, induced pluripotent stem cell (iPSC)-derived kupffer cells (iKCs) and iPSC-derived hepatic stellate cells (iHSCs). B) A powerful approach for understanding MASLD is now possible through the combination of techniques such as single-cell and spatial omics, which allow detailed analysis of gene expression, pathways characterization, genomic changes and cell-cell interactions while preserving tissue architecture [115]. Metabolic liver zonation, especially immune cell distribution, is often altered in MASLD. Spatial tissue analysis is emerging as a better approach to study zonation changes in different liver diseases [115].

### Polygenic risk scores and MASLD

Polygenic risk scores (PRS) are diagnostic tools that apply current genetic knowledge for risk stratification purposes; their success depends on the selection and combination of various risk alleles to predict a given outcome. In the case of MASLD, there have been several efforts to develop an effective PRS, but accurate prediction of a disease with such a wide spectrum can prove difficult. It is also pertinent to acknowledge that a large portion of the information on genetic risk alleles has been derived from European populations and predictions made with these data may not apply to other populations.

To address these issues, many researchers opt to focus on particular aspects or stages of this disease. Bianco et al. (2021) proposed two PRS aimed at predicting HCC risk in patients with MASLD. The PRS-HFC (Hepatic Fat Content) included four risk variants (*PNPLA3* rs738409 C > G allele, *TM6SF2* rs58542926 C > T allele, *GCKR* rs1260326 C > T allele, and *MBOAT7* rs641738 C > T allele) and PRS5 added and adjusted for a protective variant (*HSD17B13* rs72613567 allele). We have discussed the effects of these five alleles in previous sections. PRS5 performed marginally better than PRS-HFC at HCC prediction regardless of the stage of the disease [4].

 A recent study increased our understanding regarding the mechanism of action of the *PNPLA3* rs738409 G>C allele, concluding that biological aging accelerates liver fibrosis in patients with MASLD, with a clear relationship between the progression in age and the predisposition to boost liver fibrosis in presence of G/G allele for each period of 10 years [95]. Establishing clear clinical parameters — such as age and time intervals — to characterize liver damage could therefore improve patient follow-up.

Inspired by the previous study, Thomas et al. (2022) used PRS-HFC in an East Asian cohort while also developing a PRS specifically tailored for this population (PRS-NAFLD which included *GCKR* rs1260326 C > T allele, *GATAD2a* rs4808199, and *PNPLA3* rs2896019) [116]. This study aimed to assess the performance of a PRS in a population that differs from the one it was initially intended for. Results were similar for both PRS applied to an East Asian cohort, both predicted HCC risk regardless of MASLD stage [116].

The GOLDPlus GWAS meta-analysis identified seventeen risk variants for MASLD, including *PNPLA3* rs738409 C > G allele, *TM6SF2* rs58542926 C > T allele and *GCKR* rs1260326 C > T allele, and new variant alleles for *MBOAT7* and *GPAM* [117]. Chen et al. (2023) included these seventeen risk alleles in their PRS which they then applied to an American cohort of 51,550 patients (48,529 controls and 3,021 cases), individuals that scored in the top 5% of the PRS had a high risk for developing MASLD and late-stage complications for this disease (OR = 2) [117]. The authors admitted that one of the biggest limitations of their study is that most of their data come from European patient cohorts, which may affect the performance of their PRS in other populations [117]. It is of paramount importance to study populations with different ancestries to create tools that can accurately predict and stratify the magnitude of this disease around the world.

### Gene-Environment interactions for MASLD modulation

The magnitude of the effect of an allele variant greatly depends on its environmental context, which can enforce or inhibit a gene’s inductive or protective effects and influence its epistatic relationships. We have provided several examples of this interdependence, but we will delve deeper into some of the interactions between genes and environmental factors that we deem important. The magnitude of the effect of *PNPLA3* rs738409 C > G allele has a directly proportional dependence on body fat for both pathogenic and protective effects. Mild effects were observed for hepatic steatosis in allele carriers with a BMI less than 25 kg/m compared to the general population. Homozygous carriers of this allele with a BMI greater than 35 kg/m exhibited a three-fold increase in hepatic triacylglyceride content (a two-fold increase in hepatic steatosis diagnosis) compared to non-carriers [118]. Furthermore, cirrhosis risk was also observed to be higher in homozygous patients with severe obesity (OR = 5.8, BMI > 35 kg/m) compared to leaner patients (OR = 2.4, BMI < 25 kg/m) [118]. Protection against CVD varied accordingly, homozygous carriers for this allele had lower serum levels of VLDL and LDL, smaller VLDL particles, and larger LDL particles with a lower content of TG [20]. Similar effects were observed for the interaction of this allele with insulin resistance as described in previous sections.

Direct body fat dependence for risk alleles *GCKR* rs1260326 C > T and *TM6SF2* rs58542926 C > T was observed in American patients. There was a statistically significant increase in the hepatic triacylglyceride content in both homozygous and heterozygous carriers of either allele with a BMI greater than 30 kg/m [118]. Further research is needed to strengthen the evidence for these interactions in other populations. As previously mentioned, the pathogenic effects of *GCKR* rs1260326 C > T allele can also be amplified by hyperglycemia and T2DM. Body fat can also amplify the pathogenic potential of *GPAM* rs10787429 and *SERPINA1* rs28929474 alleles. In the case of *GPAM* allele, patients with a BMI greater than 30 showed elevated serum AST and ALT levels (independent of allele dose) than their leaner counterparts [44]. Patients with the *SERPINA1* rs28929474 heterozygous genotype showed a direct correlation between increased ALT and AST levels and BMI [52]. Additionally, allele carriers also showed an 8x increased probability of having a liver stiffness measurement of over 7.1 when presenting with obesity and diabetes [55].

A similar effect was observed between *GPAM* rs10787429 T > C carriers and elevated weekly alcohol consumption [44]. The protective effects of alleles in *MTARC1* and *HSD17B13* are also amplified directly by body adiposity, where patients with elevated BMI presented a lower degree of hepatic steatosis than leaner patients [68, 80]. It is important to note that most of these studies were performed in European descent populations and that environmental factors (such as comorbidities, sedentarism, and lifestyle choices) vary widely among different populations. Therefore, future studies must aim to interrogate the gene-environment interactions for different populations to add to our understanding of this condition.

MASLD has been proposed to be subdivided into “metabolic MASLD” (MM) and “genetic MASLD” (GM). This classification is based on a metabolomic study in which patients without mutations associated with increased disease susceptibility were categorized as MM, whereas those carrying at least one predisposing mutation were classified as GM. Clinically, no significant differences were observed between MM and GM groups; however, metabolomic analyses revealed a higher risk of fibrosis development in the GM group, suggesting that genetic background may serve as an early indicator for fibrosis diagnosis [119]. This study demonstrates the growing importance of considering genetic variants and the epistatic relationship between some core genes into diagnosis, staging, and treatment of MASLD.

 Sexual dimorphism and aging have historically been underrepresented in the literature, not only in MASLD research but across most pathologies. Although existing clinical and experimental models are becoming more inclusive, the impact of these factors on MASLD and epistasis modulation remains insufficiently characterized. In this context, most experimental models have focused on hypercaloric diet-induced liver dysfunction. While MASLD is more prevalent in men, women face increased risk after menopause, with an inversion in epidemiological incidence rates — a pattern also observed in type 2 diabetes (T2DM), cardiovascular conditions, and mental disorders [120].

 Estrogen receptors (ERs) display different expression levels throughout a woman's life. Epidemiological evidence shows that the incidence of various conditions, including MASLD, increases after menopause, associated with the loss of ER-mediated protection [121]. ER-α is generally considered as a protective factor against liver damage, however, female carriers of the PNPLA3 I148M variant are more susceptible to developing hepatic problems than males, regardless of age [122]. Finally, differential expression of the estrogen receptor 1 (*Esr1*) and estrogen receptor 2 (*Esr2*) genes in male and female mice fed hypercaloric diets showed that fatty liver disease in males correlates with the physiological overexpression of *Esr2*, while females with healthy liver conditions showed normal expression of *Esr1*. This suggests a potential therapeutic target for reversing diet-induced profibrotic changes in the liver [122].

 To date, clinical therapies have focused on hormone replacement therapy (HRT), but the impact of HRT on MASLD in postmenopausal women remains poorly understood. Currently, no specific therapeutic strategies exist to prevent or reverse MASLD development in menopausal women [123]. We expect that with the advent of more inclusive models, precision medicine will be able to address sexual dimorphism and improve diagnosis and treatment of women.

Although it is gradually improving, many large repositories and biobanks still contain a low number of non-European samples, complicating precision medicine efforts. As a significant example, the PanCancer Analysis of Whole Genomes (PCAWG) is one of the largest comparative population studies, reporting the integrative analysis of 2,658 whole-cancer genomes and their matching normal tissues across 38 tumour types. Demographically 93% of samples came from two regions (European countries 77%, and 16% from Asia) [124, 125]. If this lack of diversity is addressed, the increasingly large biobanks of NGS data could have a significant impact on the study and development of tools to improve the diagnosis, treatment, and prediction of drug action in many different maladies.

Artificial intelligence (AI) is emerging as a powerful non-invasive diagnostic tool, although its application in this field is still under development. As an example of the growing use of AI in this area, Zhang et al. [126] compared the efficiency of machine learning (ML) versus deep learning (DL) — the two most widely used computational approaches for diagnosing MASH and associated liver fibrosis. Through a meta-analysis of studies sourced from PubMed, Web of Science, Embase, and the Cochrane Library, comparing clinical outcomes against the predictive power of DL and ML, the authors concluded that both algorithms perform comparably to clinical diagnosis, opening significant opportunities for computational modeling as a faster and more cost-effective tool for diagnosis, treatment, and drug discovery [126].

Coupled with the potential of AI, randomized controlled trials (RCTs) remain the gold standard for evaluating medical interventions. The integration of polygenic scores for various conditions, including MASLD, has been shown to increase the statistical power of trial emulation by incorporating genetic background [127]. Taken together, increasing diversity in biobanks, broader genetic representation, and AI integration represent a powerful convergence of approaches with the potential to advance truly personalized medicine for patients.

## Conclusions

MASLD is a multifactorial entity in which both lifestyle and the presence or absence of specific genetic variants play a key role in its development. Next-generation sequencing technologies have allowed us to uncover the role that genetic background can have in this pathology. Naturally, genes and their variants do not exist in a vacuum; they interact with each other and with their environment. These interactions serve to accelerate or delay the development of this disease. It is therefore imperative to understand the mechanisms behind these interactions, ideally, this may lead to the development of a diagnostic tool that can take into consideration both genetic and lifestyle factors to calculate individual MASLD risk. Crucially, several of the variants that affect MASLD risk also impact CVD risk, which will need to be taken into account when designing therapeutic interventions informed by genetics. A thorough understanding of these interactions will be the turning point for the development of personalized treatments which could help to decrease the burden that this disease will exert on healthcare systems in the coming years.

## Data Availability

No datasets were generated or analysed during the current study.

## Abbreviations

AI: Artificial intelligence
A1AT: Alpha 1-antitrypsin
A1ATD: Alpha 1-antitrypsin deficiency
AASLD: American association for the study of liver diseases
AF: Allele frequency
ALD: Alcoholic liver disease
ALEH: Latin American association for the study of the liver
ALP: Alkaline phosphatase
ALT: Alanine aminotransferase
AST: Aspartate aminotransferase
BMI: Body mass index
CVD: Cardiovascular disease
DL: Deep learning
EASL: European association for the study of the liver
FDA: Food and drug administration
GM: Genetic MASLD
GK: Hepatic glucokinase
GKRP: Glucokinase regulatory protein
GWAS: Genome-wide association studies
HCC: Hepatocellular carcinoma
HFC: Hepatic fat content
HOMA-IR: Homeostatic model assessment for insulin resistance
HRT: Hormone replacement therapy
HSCs: Hepatic stellate cells
IL-6: Interleukin 6
iPSCs: Induced pluripotent stem cells ()
LAMPS: The liver acinus microphysiology system
LDL: Low-density lipoprotein
LoC: Liver-on-a-chip
MASH: Metabolic dysfunction-associated steatohepatitis
MASLD: Metabolic dysfunction-associated steatotic liver disease
ML: Machine learning
MM: Metabolic MASLD
MPS: Microphysiological systems
NAFLD: Non-alcoholic fatty liver disease
NASH: Non-alcoholic steatohepatitis
OoC: Organoid on a chip
OR: Odds ratio
PCAWG: PanCancer analysis of whole genomes
PRS: Polygenic risk scores
PRS-HFC: Polygenic risk score-hepatic fat content
RCTs: Randomized controlled trials
SNP: Single-nucleotide polymorphism
T2DM: Type 2 diabetes mellitus
TAM: Tumour-associated macrophage
TC: Total cholesterol
TG: Triacylglycerides
VLDL: Very low-density lipoprotein
